# Immuno-Acoustic Sorting of Disease-Specific Extracellular Vesicles by Acoustophoretic Force

**DOI:** 10.3390/mi12121534

**Published:** 2021-12-09

**Authors:** Junyuan Liu, Yuxin Qu, Han Wang

**Affiliations:** Department of Biomedical Engineering, School of Medicine, Tsinghua University, Beijing 100084, China; hljljy305@126.com (J.L.); qyx20@mails.tsinghua.edu.cn (Y.Q.)

**Keywords:** extracellular vesicle, acoustophoresis, immunoaffinity, HER2

## Abstract

Methods for the isolation and analysis of extracellular vesicles (EVs) have been extensively explored in the field of life science and in clinical diagnosis in recent years. The separation and efficient recovery of high-purity target EVs from biological samples are important prerequisites in the study of EVs. So far, commonly used methods of EV separation include ultracentrifugation, filtration, solvent precipitation and immunoaffinity capturing. However, these methods suffer from long processing time, EV damage and low enrichment efficiency. The use of acoustophoretic force facilitates the non-contact label-free manipulation of cells based on their size and compressibility but lacks specificity. Additionally, the acoustophoretic force exerted on sub-micron substances is normally weak and insufficient for separation. Here we present a novel immuno-acoustic sorting technology, where biological substances such as EVs, viruses, and biomolecules, can be specifically captured by antibody/receptor coated microparticles through immunoaffinity, and manipulated by an acoustophoretic force exerted on the microparticles. Using immuno-acoustic sorting technology, we successfully separated and purified HER2-positive EVs for further downstream analysis. This method holds great potential in isolating and purifying specific targets such as disease-related EVs from biological fluids and opens new possibilities for the EV-based early diagnosis and prognosis of diseases.

## 1. Introduction

The detection and analysis of extracellular vesicles (EVs) have been topics of intensive research in the fields of life science and clinical diagnosis in recent years. Based on size and biogenesis, extracellular vesicles can be classified into three types, including exosomes (40–120 nm), microvesicles (50–1000 nm) and apoptotic bodies (>1000 nm) [1]. Containing rich amounts of intracellular molecules (e.g., messenger RNAs, microRNAs and proteins) and membrane proteins, EVs hold biochemical cues of their producing cells, providing valuable information about related diseases [1,2,3,4,5]. Compared with other disease biomarkers, e.g., circulating tumor cells, EVs are abundant in quantity [6], thus making them more convenient to be collected and analyzed. In addition, a protective phospholipid bilayer keeps their inner biomolecules from being decomposed, making EVs more stable than circulating cell-free nucleic acids and proteins. Numerous studies have proved the potential clinical utility of EVs in the diagnosis of cancer [7], acquired immunodeficiency syndrome (AIDS) [8], Alzheimer’s disease [9], and Parkinson’s disease [10], etc. Therefore, EVs are ideal biomarkers that can be collected and analyzed by liquid biopsy for early screening, diagnosis and prognosis of diseases.

In order to study EVs, the important prerequisites are efficient isolation and recovery of target EVs from biological samples. Current isolation methods include ultracentrifugation, filtration, solvent precipitation and immunoaffinity capturing, etc. [11]. Ultracentrifugation is the most commonly used technique to isolate and purify EVs. However, it requires time-consuming operation, causes contamination with protein aggregates and results in low EV-associated markers [12,13]. Moreover, high centrifugal force has been shown to lead to EV fusion and coagulation, which may alter their structures and functions [6]. Filtration, a common method for separating particles based on their sizes, can also be used to isolate EVs [11]. It works faster than ultracentrifugation but has drawbacks, such as being prone to clogging and physically damaging EVs during separation. Other conventional methods, such as solvent precipitation [14] and immunoaffinity capturing [13], are easier and faster than ultracentrifugation but suffer from low recovery efficiency.

Microfluidic technology offers compelling advantages in the separation, manipulation and concentration of biological particles [15]. Utilizing the merits of microfluidics, researchers have explored various methods to isolate EVs based on their physical properties, including microfluidic filtering, deterministic lateral displacement (DLD) sorting, viscoelastic flow sorting, etc. [16]. However, these methods cannot isolate EVs specifically or eliminate contaminants such as protein aggregates efficiently. Some researchers have combined immunoaffinity with microfluidics to isolate EVs. Chen et al. [17] presented a microfluidic method of isolating EVs from serum by modifying certain antibodies on the surfaces of their microchannels, enabling fast and specific EV separation from a small amount of sample. However, the amount of EVs isolated by this method was limited by the area of the microchannel surface, leading to insufficient EV recovery. In addition, devices like ExoChip [18], iMER [19], ExoSearch [20] and Nano-IMEX [21] also utilize the merits of microfluidic platforms and immunoaffinity to isolate EVs, but these methods still suffer from limitations in isolation throughput and recovery efficiency. In order to improve the performance of microfluidic devices, microchannels can be integrated with different forces, such as fluid force, optical force, electromagnetic force and acoustophoretic force, to precisely process complex biological samples on the micro–nano scale. The acoustofluidic technique is based on the integration of microfluidics and acoustophoretic force. Acoustophoretic force, produced by piezoelectric transducer (PZT), is a contactless field force that is proportional to the particle’s biophysical properties. Thus, the acoustofluidic technique has been widely utilized in the separation or manipulation of cells and other nano/microparticles [22,23,24,25,26]. For instance, Cui et al. [25] presented hypersonically induced 3D hydrodynamic tweezers to manipulate micro/nanoscale objects versatilely. Additionally, surface acoustic waves [26] or ultralow frequency acoustic vibration [23] have been used for producing micro-vortices to facilitate nanoparticle manipulation. To overcome the issues with conventional EV-isolating methods, Wu et al. [6] demonstrated an acoustofluidic platform to separate EVs directly from undiluted blood based on their sizes. Their platform includes a cell-removal module and an EV-isolation module, facilitating the removal of larger blood components, initially, followed by the separation of EVs. This approach improves the efficiency of EV isolation. However, limited by wavelength, the acoustic force cannot separate EVs from protein aggregates or other non-exosomal nanoparticles, leading to low purity. Additionally, this method relies on physical separation by surface acoustic waves, which cannot isolate target EVs that are related to specific diseases. Overall, there is an urgent need to develop a system that can directly process biological samples to separate target EVs specifically and recover them efficiently.

Here, we propose a novel immuno-acoustic sorting technology, where the specific capturing of target EVs by immunoaffinity and the contactless manipulation of captured EVs by the acoustophoretic force are combined to realize the specific separation, washing and recovery of disease-related target EVs. Using immuno-acoustic sorting technology, we successfully separated and purified HER2-positive EVs from cell suspension. This method holds great potential in isolating and purifying specific targets such as disease-related EVs from the whole blood samples of patients by liquid biopsy and opening up a new path for EV-based early diagnosis and prognosis of diseases.

## 2. Materials and Methods

### 2.1. Design and Working Principles of the Immuno-Acoustic Sorting Method

**Figure 1:**
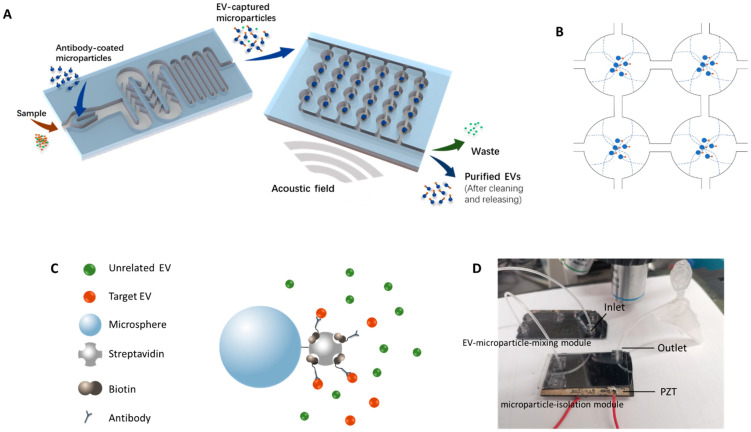
Illustration of the immuno-acoustic sorting technology. (**A**) In the EV-microparticle mixing module, the sample and antibody-coated microparticles are fully mixed, allowing the microparticles to capture the EVs. Then the mixture is added to the microparticle-isolation module, where the EV-captured microparticles are enriched in the center of the circular chamber by acoustophoretic force. After cleaning and removing the acoustic field, purified EVs can be obtained from the outlet. The solution that flows from the outlet during this time is waste. After cleaning and stopping the acoustic field, purified EVs can be obtained from the outlet. (**B**) Principle of microparticle enrichment using acoustic waves. Acoustic standing waves were generated at specific frequencies and the pressure nodes were formed in the center of the chambers, driving the EV- microparticle conjugates to move towards the center of the chambers. (**C**) Modification of the microparticles. The target EVs is captured by the antibodies modified on the microparticles utilizing streptavidin-biotin system (Figure S1). (**D**) Photographic image of the device.

In this work, a new method was established to capture target EVs by antibody-coated microparticles and to collect the EV-captured microparticles for further analysis. Immuno-acoustic sorting technology was comprised of an acoustofluidic microchip consisting of two major modules (Figure 1A), the EV-microparticle mixing module and the EV-microparticle conjugate isolation module. The EV-microparticle mixing module was designed to mix the samples with antibody-coated microparticles, allowing target EVs to fully interact with them and be captured. In the EV-microparticle conjugate isolation module, EV-microparticle conjugates were exposed to acoustophoretic force and separated for downstream analysis.

#### 2.1.1. Working Principle of the EV-Microparticle Mixing Module

The schematic of the working principle for EV-microparticle mixing is shown in the left side of Figure 1A. The sample and the antibody-coated microparticles are added to the mixing module simultaneously, followed by mixing in the channel with baffles. After that, the mixture is incubated in a long, serpentine channel for antigen–antibody binding.

#### 2.1.2. Working Principle of the Acoustophoretic Force

To enrich the EV-captured microparticles, a piezoelectric transducer (APC 841, APC International, Ltd., Mackeyville, PA, USA) was attached underneath the microchip by a cyanoacrylate adhesive to generate the acoustic resonance field in the chip. The footprint of the PZT was 25 mm × 50 mm × 2.2 mm. Acoustic fields can be formed by applying sinusoidal signals to the PZT. In order to utilize the acoustophoretic force for the enriching and washing of EV-captured microparticles, circular chambers were designed to form acoustic standing waves and capture microparticles. When the half-wavelength of the acoustic wave equals the diameter of the circular chamber, acoustic standing waves are generated and the pressure nodes form in the center of the chambers. As a result, the microparticles inside the circular chamber move towards the center of the chamber by acoustophoretic force. The acoustophoretic force is described by Equations (1) and (2) [27].

(1) $$F_{a}=-\left(\frac{\pi p^{2}V_{0}\beta_{s}}{2\lambda }\right)\Phi \left(\beta ,\rho \right)\sin(2ky)$$

(2) $$\Phi \left(\beta ,\rho \right)=\frac{5\rho_{0}-2\rho_{s}}{2\rho_{0}+\rho_{s}}-\frac{\beta_{0}}{\beta_{s}}$$

where *V*_0_ represents the volume of the particle, *p* is the acoustic pressure amplitude, *λ* is the wavelength of the acoustic field, *ρ*_0_ is the density of the particle, *ρ_s_* is the density of the solution, *β*_0_ is the compressibility of the particle, and *β_s_* represents compressibility of the fluid. Equation (2) shows the expression of an acoustic contrast factor *Φ*(*β*, *ρ*), which depends on the physical properties of the microparticles and the solution, that determines the direction that the particles move. The *Φ*(*β*, *ρ*) of the microparticles in the solution exhibited positive values in this study, indicating that they were prone to move towards the acoustic pressure nodes in the acoustically resonant field (Figure 1B).

The resonance frequency was calculated first by *λ* = *v*/*f*, where *λ* stands for the wavelength of the acoustic field, *v* stands for the velocity of the wave and *f* is the calculated resonance frequency. To generate the first order acoustic resonance field, the wavelengths of acoustic waves should be equal to twice the diameter of the microchambers. Due to the limitations of fabrication precision, the optimized resonance frequency was not exactly the same as the calculated resonance frequency. As a result, the optimized frequency was determined prior to testing with EVs by tuning the frequency of the sinusoidal signal around the calculated resonance frequency to generate the first-order resonance field that could capture EV-microparticle conjugates in the pressure node of the circular chamber. In the EV-microparticle conjugate isolation module, the inlet flow rate was set at 0.5 mL/h, while the amplitude of the sinusoidal signal on the PZT was 400 mV and the frequency was 537 kHz.

### 2.2. Fabrication and Operation of the Microchip

Fabrication of the microchip followed previously described procedures [28]. In brief, the microchip was made of a silicon wafer and borofloat glass. Microfabrication techniques were used to fabricate the microchannels for EV manipulation on the silicon substrate. We used a 3-µm-thick AZ4620 photoresist to form patterns by photolithography and etched microchannels by deep reactive ion etching (SPTS OMEGA LPX RAPIER, SPTS, Shanghai, China). The depth of the microchannels was 50 µm. In the EV-microparticle mixing module, 300 μm-wide serpentine channels with baffles were designed to facilitate complete mixing, followed by the creation of 200 μm-wide serpentine channels for incubation. In the EV-microparticle conjugate isolation module, 51 circular chambers with the diameter of 1.5 mm were designed. The microfluidic channels connecting the chambers were 200 μm in width.

A drill press was used to drill fluidic access holes in the borofloat cover glass. After cleaning the silicon wafer and the cover glass, anodic bonding was conducted to bond them together. The tubing was attached to the microchip for fluid inflow/outflow by insertion into a polydimethylsiloxane (PDMS) block bonded on the cover glass. Wax was used to glue the piezoelectric transducer to the bottom of the microchip. The photo of the device is shown in Figure 1D.

### 2.3. Modification of the Microparticles

To modify the microparticles, 80 μL 1.25% streptavidin-coated polystyrene microparticles (Polysciences, Warrington, PA, USA) were centrifugated at 10,000× *g* for 5 min and washed by PBS/BSA binding buffer for 3 times. After removing and discarding the supernatant, 100 μL 0.2 mg/mL Human ErbB2/Her2 Biotinylated Antibody (R&D Systems, Minneapolis, MN, USA) was incubated with the microparticles for 30 min to fabricate HER2 antibody-coated microparticles (Figure 1C), followed by washing three times. Finally, 100 μL PBS/BSA binding buffer was added to resuspend the modified microparticles.

### 2.4. Cell Line Culture and EV Preparation

The human breast cancer cell lines BT474 and MDA-MB-231 were purchased from the National Infrastructure of Cell Line Resource (Beijing, China) and cultured according to the instructions. Specifically, MDA-MB-231 cell lines were maintained in the DMEM medium (Thermo Fisher Scientific, Waltham, MA, USA) supplemented with 10% (*v*/*v*) fetal bovine serum (FBS) and 100 U·mL^−1^ penicillin-streptomycin. BT474 cell lines were grown in RPMI-1640 medium (Thermo Fisher Scientific, Waltham, MA, USA) with 10% (*v*/*v*) FBS, 100 U/mL penicillin-streptomycin and 0.01 mg/mL insulin (Macklin Inc., Shanghai, China). Both of the cells were cultured in the cell incubator with 5% CO_2_ at 37 °C. The FBS was prepared by ultracentrifugation at 100,000× *g* for 18 h, the supernatant was separated for medium preparation to exclude the interference of extracellular vesicles from FBS.

For verification experiments of the immuno-acoustic sorting technology, EVs were isolated from cell suspension by ultracentrifugation to ensure the sufficiency of target EVs. The cell suspension was centrifugated at 3000× *g* (4 °C) for 30 min to remove cell debris, then the supernatant was transferred to new tubes and spun at 10,000× *g* (4 °C) for 30 min to pellet other large particles. The final supernatant was gently transferred to Ultra-Clear Tubes (26.3 mL, Beckman Coulter Inc., Brea, CA, USA) by Pasteur pipette, followed by ultracentrifugation at 100,000× *g* (4 °C) for 70 min. After discarding the supernatant, the precipitation was resuspended with PBS and spun at 100,000× *g* (4 °C) for 70 min to wash the EVs. Finally, 400 µL PBS was added to the Ultra-Clear Tubes to collect the concentrated EV pellets.

### 2.5. Western Blot Analysis and TEM Imaging of the Samples

Western blot analysis was performed to detect the specific proteins in the cells and the EVs. The cells were lysed in RIPA lysis buffer (Beyotime, Shanghai, China) with 1% phenylmethylsulfonyl fluoride (PMSF, Beyotime, Shanghai, China) mixture. For the EVs, 1% Nonidet P-40 (Beyotime, Shanghai, China) with 1% cocktail (Roche, Basel, Switzerland) were added for brief lysis. Both of the samples were heated at 95 °C for 10 min after addition of loading buffer and loaded on SDS–polyacrylamide gel electrophoresis (PAGE, Biotides, Beijing, China). The electrophoresis was carried out at 80 V for 20 min first and then run at 120 V for 40 min. After that, the proteins on the gels were electrotransferred to the nitrocellulose membrane at 200 mA for 2 h. Then nitrocellulose membrane was blocked by 5% skim milk in TBST buffer, followed by incubation in primary antibody (anti-HER2, anti-CD44, anti-GAPDH antibodies at the concentration of 1:1000 in 5% skim milk) overnight at 4 °C. After TBST washing, the membrane was placed in HRP-conjugated secondary antibody (1:10,000 in TBST) for 1 h. Imaging of the blotting results was performed after adding the chemiluminescence substrate (Thermo Fisher Scientific, Waltham, MA, USA).

To characterize the morphology of the EVs, transmission electron microscopy (TEM) imaging was carried out. The concentrated EVs were dropped on 400-mesh copper grids and stained in uranyl acetate, followed by TEM imaging.

### 2.6. EV Capture Using Microparticles

The purpose of the EV-microparticle mixing step was to blend the antibody-coated microparticles with the cell suspension samples, forming EV-microparticle conjugates. Baffles were designed inside the microchannels in order to form turbulence, which increased the contact between the EVs and the microparticles and improved the capture efficiency. Considering the low concentration of the EVs in the original cell suspension, the cell suspension was concentrated by ultracentrifugation to ensure the sufficiency of target EVs. The concentrated EV pellets of BT474 and MDA-MB-231 were diluted from 30 µL to 250 µL by PBS as the input samples separately. For the EV capture test using microparticles, we used two Harvard syringe pumps (Harvard Apparatus, Holliston, MA, USA) to control the flow rates of the EV sample and the microparticle solution, which were set to be 0.5 mL/h and 0.04 mL/h, respectively. The total time for mixing and incubation was 30 min. Then both of the injected samples were changed into PBS, which was injected at 0.5 mL/h for 20 min to clean the microchannel. The EV-microparticle conjugates were collected in the outlet.

### 2.7. Characterization of Capture Efficiency and Specificity of the EVs

Scanning electron microscopy (SEM) was used to characterize the morphology of the bare, EV-captured and after-lysis microparticles. Bare microparticles and HER2 antibody-coated microparticles were mixed with BT474 EV samples separately, and centrifugated at 10,000× *g* to obtain the precipitates. The two precipitates were denoted by Bare microparticles and EV-captured microparticles, respectively. For EV-captured microparticles, some of the microparticles were briefly lysed by 1% Nonidet P-40 (Beyotime, Shanghai, China) with 1% cocktail (Roche, Basel, Switzerland) and centrifugated to get the precipitates as after-lysis microparticles. These three samples were prepared for scanning electron microscopy (SEM) imaging. To visualize the surfaces of the microparticles, the samples were fixed in 2% paraformaldehyde and 2.5% glutaraldehyde for 2 h. Then for dehydration, 50%, 70%, 80%, 90% and 100% ethanol solutions were used to immerse the samples for 5 min, one after another. After that, samples were lyophilized overnight, and a thin layer of gold was sputtered to the surface of the microparticles for SEM imaging.

For both BT474 and MDA-MB-231 EVs, the concentrations and the size distributions before and after mixing with the microparticles were tested by nanoparticle tracking analysis (NTA). The samples before mixing and the supernatant after mixing were diluted to 3 mL and tested with a NanoSight LM10 instrument, respectively.

For characterization of the capture specificity of EVs, enzyme linked immunosorbent assay (ELISA) was performed. For sample loading, the precipitates of mixed samples were lysed by 100 µL 1% Nonidet P-40 (Beyotime, Shanghai, China) with 1% cocktail (Roche, Basel, Switzerland), while the supernatants were concentrated by protein concentrators (Thermo Fisher Scientific, Waltham, MA, USA) first and then lysed under the same condition. For samples that had mixed with BT474 EVs, the final concentrations of HER2 in the loading samples of the precipitates and the supernatants were tested using Human ErbB2/HER2 ELISA Kit (Thermo Fisher Scientific, Waltham, MA, USA). Specifically, the samples were transferred to a 96-well plate and incubated for 2.5 h at room temperature with gentle shaking. After washing with washing buffer three times, 100 µL biotinylated HER2 antibody, at the appropriate concentration, was added to each well and incubated for 1 h. Then unbounded biotinylated HER2 antibody was triple-washed with washing buffer, followed by 45-min incubation with HRP-streptavidin and washed three times. Lastly, 100 µL ELISA Colorimetric TMB Reagent was added to each well. After 30 min, 50 µL stop solution was added and the signal was read at 450 nm immediately. For the samples that had mixed with MDA-MB-231 EVs, standard ELISA procedure was conducted using a Human CD44 ELISA Kit (Sigma-Aldrich, Copenhagen, Denmark) to detect the final concentrations of CD44 in the loading samples. The samples were incubated for 1 h followed by washing three times, then biotinylated anti-CD44 were added to each well for a 30-min incubation. After that, HRP-streptavidin solution was added and incubated for 30 min. Then 100 µL TMB reagent was added to each well after washing. Finally, the absorbance was measured at 450 nm and 620 nm.

### 2.8. EV-Microparticle Conjugate Enrichment Using Acoustophoretic Force

After treatment by 2% F-108 (Sigma-Aldrich, Copenhagen, Denmark), ethanol was injected into the microchannels to exhaust air. Then the inlet solution was changed to PBS to remove the ethanol and make sure no bubbles had been generated during this process. After PBS filled the channel, the EV-microparticle conjugates solution was aspirated into a syringe and perfused at the flow rate of 0.5 mL/h. A sinusoidal signal at the frequency of 537 kHz and the amplitude of 400 mV was generated by a signal generator (RIGOL Technologies, Beijing, China), and amplified by a 50 dB RF power amplifier (Bell Electronics, WA). The amplified signal was applied to the PZT prior to sample injection. During this time, the conjugates were captured in the microfluidic chambers. After 24 min, PBS buffer was introduced into the microchannel to wash out free microparticles and contaminants in the original solution. During conjugate capturing and PBS cleaning, the solution collected at the outlet was waste. After cleaning, the sinusoidal signal was removed and PBS was injected at 1 mL/h for 30 min to flush out the captured EV-microparticle conjugates, which were thereby collected at the outlet.

Before the EV capture experiments, microparticles with diameters of 3 µm and 6 µm were tested in order to determine the optimized size of the microparticles. For both types of microparticles, 25 mg/mL polystyrene microparticles (Tjdaekj, Tianjin, China) were diluted 500-fold, 1000-fold and 1500-fold, each, for testing at a final volume of 250 μL by PBS. The microparticle solutions were spiked into the circular chambers at 0.5 mL/h under an acoustic resonance field, separately, followed by PBS cleaning and microparticle flushing. To quantify the number of the microparticles lost or collected, a cell counting plate (Bio-Rad, Hercules, CA, USA) was used to measure the concentration of microparticles in the waste and the collected EV-microparticle conjugates, respectively, and the total numbers were calculated by multiplying the concentration and the volume. The lost/got numbers were calculated respectively.

After capturing EVs and collecting the EV-microparticle conjugates, the microparticles were lysed and the concentration of HER2 was tested using a Human ErbB2/HER2 ELISA Kit.

## 3. Results and Discussion

### 3.1. Antigen Validation in Cells and EVs

**Figure 2:**
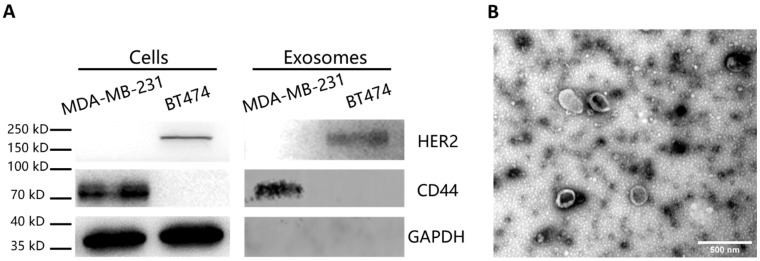
Characterization of the EV samples. (**A**) Western blot with expression of MDA-MB-231 marker (CD44), BT474 marker (HER2) and loading control (GAPDH) in cells and EVs. (**B**) TEM image of the EVs collected by ultracentrifugation.

BT474 and MDA-MB-231 cell lines were cultured and their EVs in the supernatant were isolated by ultracentrifugation. After the lysis of cells and EVs, we used Western blot analysis to evaluate the presence of particular proteins in the cells and the EVs (Figure 2A). The Western immunoblotting showed that both BT474 cells and the collected EVs from the BT474 cell suspension contained HER2 (BT474 marker) but no CD44 (MDA-MB-231 marker). In contrast, both MDA-MB231 cells and the collected EVs were immune-positive for CD44 rather than HER2. Moreover, the SEM image shows that the concentrated EVs were saucer-shaped (Figure 2B), consistent with previous studies [29].

### 3.2. Immunoaffinity Capture of EVs Using Antibody-Coated Microparticles

**Figure 3:**
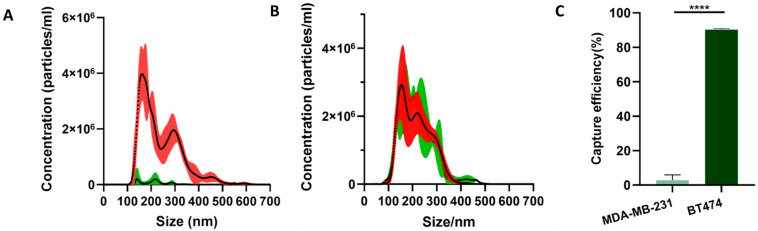
(**A**–**C**) NTA results for cell suspension of BT474 and MDA-MB-231. (**A**) Size distribution of particles before (red curves showing standard deviation) and after (green curves showing standard deviation) the BT474 suspension mixed with the HER2 antibody-coated microparticles. (**B**) Size distribution of particles before (red curves showing standard deviation) and after (green curves showing standard deviation) the MDA-MB-231 suspension mixed with the HER2 antibody-coated microparticles. (**C**) The capture efficiency of HER2 antibody-coated microparticles for BT474 suspension and MDA-MB-231 suspension, respectively. **** *p* < 0.0001.

In order to evaluate the specificity of EVs capture using antibody-coated microparticles, the diluted EV samples of MDA-MB-231, as well as those of BT474, were mixed with microparticles in the EV-microparticle mixing module, respectively. The microparticles coated with HER2 antibodies could specifically bind to HER2-positive EVs from the suspension of BT474 culture rather than that of MDA-MB-231. To confirm that the target EVs could be captured by HER2 antibody-coated microparticles while unrelated EVs could not, we conducted an NTA to quantify the EVs in the samples before and after mixing with antibody-coated microparticles and calculated the capture efficiency of two different EVs. The capture efficiency of the EVs was defined as the ratio of the number of nanoparticles at 0–700 nm after mixing to that of the nanoparticles of the same size before mixing [6]. The concentration of the particles whose sizes were 0–700 nm in the original samples of BT474 cell line was calculated to be 5.19 × 10^8^ per microliter, while that after the mixing was 4.04 ± 1.74 × 10^7^ per microliter (Figure 3A). The reduction of the microparticles suggested that most nanoparticles 0–700 nm in size were attached to the microparticles. Therefore, the capture efficiency of EVs from the suspension of BT474 was calculated to be 90.21 ± 0.66%. In contrast, for the EV samples of MDA-MB-231, the concentration of particles, before mixing, with diameters in the range of 0–700 nm was 4.46 × 10^8^ per microliter, while that after mixing was 4.28 ± 0.13 × 10^8^ per microliter (Figure 3B). Thus, the capture efficiency of EVs from the suspension of MDA-MB-231 was 4.15%. These results demonstrated that the antibody-coated microparticles can specifically capture EVs through immunoaffinity.

**Figure 4:**
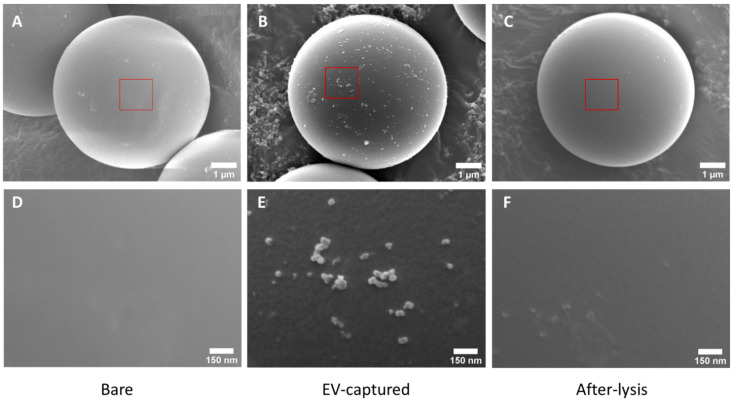
Morphological characterization of the bare, EV-captured and after-lysis microparticles. (**A**,**B**) are SEM images of a bare microparticle (**A**) and a HER2 antibody-coated microparticle (**B**) that had mixed with BT474 EV samples. (**C**) is the SEM image of the EV-captured HER2 antibody-coated microparticle after lysis. (**D**–**F**) show the enlarged view of the red box regions in (**A**–**C**).

We further examined the morphology of the microparticles by SEM. Bare streptavidin-coated microparticles without HER2 antibody coating and the HER2 antibody-coated microparticles were both mixed with microparticles in the EV-microparticle mixing module. The SEM images show that bare microparticles had few EVs attached to them (Figure 4A,D), while HER2 antibody-coated microparticles had captured quite a lot of EVs (Figure 4B,E). Approximately 600 vesicles were attached to the surface of a HER2 antibody-coated microparticle with the size of 30–500 nm, which was consistent with the size of EVs. The EV-captured microparticles were then lysed and imaged by SEM as well (Figure 4C,F), demonstrating that the vesicles captured by the microparticles had been lysed sufficiently.

### 3.3. Enrichment of EV-Microparticle Conjugates

**Figure 5:**
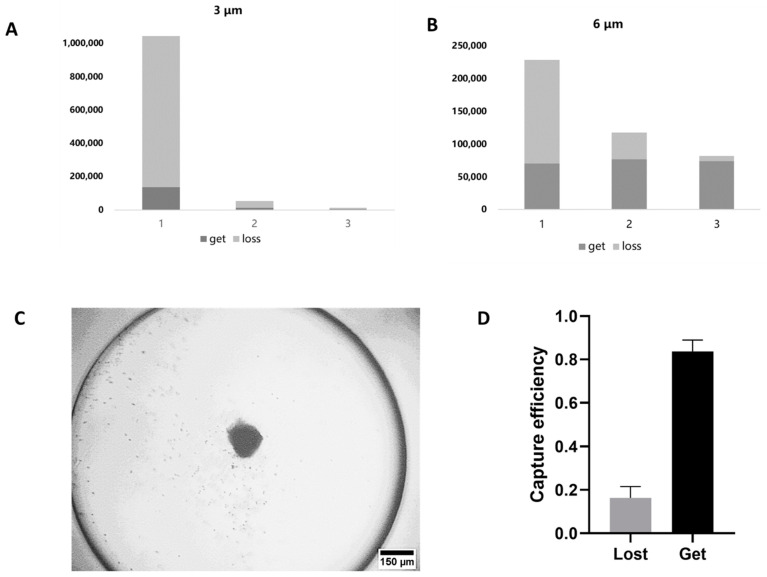
Verification of the microparticle-isolation module. (**A**,**B**) The number of microparticles with the diameter of 3 μm (**A**) and 6 μm (**B**) got and lost by acoustic resonance field at three different concentrations, 1: high concentration, 6 × 10^5^/mL for microparticles with the diameter of 6 μm, 1 × 10^6^/mL for microparticles with the diameter of 3 μm; 2: medium concentration, 4 × 10^5^/mL for microparticles with the diameter of 6 μm, 1 × 10^4^/mL for microparticles with the diameter of 3 μm; 3: low concentration, 2 × 10^5^/mL for microparticles with the diameter of 6 μm, 5 × 10^4^/mL for microparticles with the diameter of 3 μm). (**C**) Microscopic image of the enriched microparticles in the circular chamber. (**D**) Capture efficiency of the 6-μm microparticles in optimized concentration.

An optimized driving frequency of 537 kHz was identified based on the pre-experiments. The microparticles can be concentrated into the center of the circular chamber in the acoustic resonance field (Figure 5C).

In order to optimize the size and concentration of the microparticles to be used for immunoaffinity capture, particles with the diameters of 6 μm and 3 μm at three different concentrations were perfused into the circular chamber under an acoustic resonance field, as mentioned above. The number of microparticles that were got or lost was statistically analyzed, the results of which are shown in Figure 5A,B. For microparticles with the diameter of 6 μm, 7.05 × 10^4^, 7.70 × 10^4^ and 7.38 × 10^4^ microparticles were trapped in the centers of the circular chambers, 1.58 × 10^5^, 4.08 × 10^4^ and 7.65 × 10^3^ microparticles were lost. For microparticles with the diameter of 3 μm, 1.35 × 10^5^, 1.17 × 10^4^ and 3.86 × 10^3^ microparticles were trapped in the centers of the circular chambers, and 9.09 × 10^5^, 4.11 × 10^4^ and 8.27 × 10^3^ microparticles were lost. Larger microparticles suffered from smaller specific surface area and increased precipitation in the syringes and microchannels during the experiments; therefore, they were not utilized in the following tests.

In order to quantify the microparticle trapping results, the total surface area of the microparticles was calculated as S = N·4πr^2^, where N is the total number of the microparticles and r represents the radius of the microparticles. Both the mean number and the radius of the microparticles of 6 μm diameter are larger than that of the microparticles of 3 μm diameter. With the combination of the captured number in consideration, the microparticles of 6 μm diameter with the concentration of 5 × 10^5^/mL in 200 μL PBS (1 × 10^5^ in total) were used for the enrichment of EV–microparticle conjugates.

### 3.4. Characterization of the Performance of EVs Captures

After mixing with the diluted EV samples of both the BT474 and MDA-MB-231 cell cultures, microspheres coated with and without HER2 antibodies were isolated by centrifugation. The supernatant and precipitations were lysed and ELISA was performed, following standard procedures, to evaluate the specificity of the immunoaffinity capture. In the lysate of precipitations of the HER2 antibody-coated microparticles that had mixed with diluted EV samples of BT474, the concentration of HER2 was determined to be 0.2987 ± 0.027 ng/mL using ELISA, while the concentrated supernatant contained little HER2 (0.030 ± 0.012 ng/mL). For EV samples diluted with MDA-MB-231, 1.033 ± 0.081 ng/mL CD44 was present in the concentrated supernatant, while only 0.484 ± 0.0004 ng/mL CD44 was found in the precipitants.

**Figure 6:**
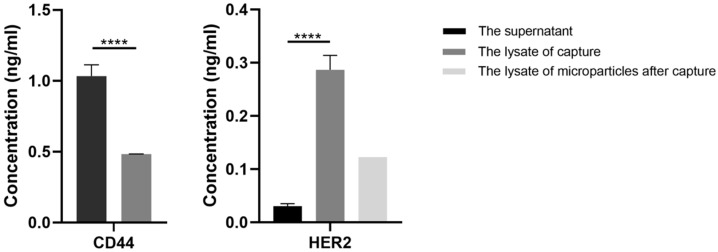
The ELISA results for CD44 and HER2 after immunoaffinity capture of EVs using HER2 antibody-coated microparticles in the supernatant and the lysate of the microparticles. For the EVs of MDA-MB-231, the concentrations of CD44 in the lysate of HER2 antibody-coated microparticles and the supernatant were tested. For the EVs of BT474, the concentrations of HER2 were tested. Additionally, the normalized concentration of the collected samples after EV-microparticle conjugate isolation is shown in the lightest gray column. **** *p* < 0.0001.

ELISA was also conducted to characterize the HER2 levels in the collected samples after the enrichment with EV-microparticle conjugates (Figure 6). The result shows that the concentration of the sample after isolation by acoustophoretic force was 0.123 ng/mL, with a percentage rate of 38.7% of the total HER2 in the loading diluted exosome solution. Compared with other EV isolation methods, such as ultracentrifugation, the main advantage of the developed immune-acoustic sorting method is that it combines the capability of acoustophoretic microparticle manipulation and the specificity of immunoaffinity capturing, so that target EVs with specific markers can be selectively captured from the analytes. Ultracentrifugation has been commonly used to collect all EV contents, however, it lacks specificity and cannot be integrated into automated microfluidic sample processing and analysis. Besides, the immune-acoustic method is highly scalable and can improve the throughput with rational designs that incorporate larger mixing channels and trapping chambers. Moreover, the samples can be introduced into the acoustofluidic device continuously, thus it is not limited to processing large quantities of samples. Though we have not tested with clinical samples yet, we believe the immune-acoustic sorting method holds great potential in clinical applications.

## 4. Conclusions

In this work, we have successfully developed a new method to isolate EVs specifically by proposing an immuno-acoustic sorting technology. This technology combines the advantages of highly specific capturing by immunoaffinity and non-contact cell manipulation capability by acoustofluidics, which facilitates the specific isolation, purification and analysis of target exosomes from biological samples using an integrated and automated microfluidic apparatus. This technology has unique advantage by integrating microfluidics with immunoaffinity and acoustofluidics, providing an effective platform for high-purity and high-specificity EV isolation. The designed microsystem in the EV-capture module achieved complete mixing and incubation of antibody-coated microparticles with the target EVs, allowing specific and efficient capture of the EVs through immunoaffinity. Using the antibody-coated microparticles as both an immunoaffinity tool and an acting intermediate material for acoustofluidic manipulation, immuno-acoustic sorting technology empowers acoustofluidic technology with the capability of the specific manipulation of biological samples. In this study, the optimized frequency and size of microparticles facilitated non-contact enrichment of the EV-captured microparticles by acoustophoretic force.

Using this technology, we have specifically captured HER2-positive EVs by the antibody-coated microparticles and enriched the microparticles in the acoustic resonance field. To improve the capture efficiency, further research needs to be done by integrating microhydrodynamics with acoustic force. Researchers have demonstrated complete mixing with acoustic waves by generating turbulence in microchannels using the oscillation of sidewall-trapped air bubbles driven by the acoustic field [30]. In future work, we will explore the feasibility of integrating acoustic mixing into a combined acoustofluidic device for EVs capture, trapping, washing and recovery. This technology opens a new possibility for EV-based early diagnosis and prognosis of diseases such as cancer. Apart from the separation of EVs, this technology can also be widely applied to the isolation and purification of other biological targets, such as cells, bacteria, viruses and biomolecules.

## Supplementary Materials

The following are available online at https://www.mdpi.com/article/10.3390/mi12121534/s1, Figure S1: Microscopic images of the enriched microparticles in the microfluidic chambers.
