# Supplementary material for: Immuno-Acoustic Sorting of Disease-Specific Extracellular Vesicles by Acoustophoretic Force
Source: Micromachines (Basel). 2021 Dec 9;12(12):1534. doi: 10.3390/mi12121534 (PMC8709371; doi:10.3390/mi12121534)
Supplement: Supplementary file 1 [file micromachines-12-01534-s001.zip › micromachines-1490956-SI.pdf]

Supplementary Materials for

Immuno-acoustic sorting of disease-specific extracellular vesicles by acoustophoretic force

Junyuan Liu[1†], Yuxin Qu[1†] and Han Wang[1,]*

[1]Department of Biomedical Engineering, School of Medicine, Tsinghua University, Beijing 100084, China; hljljy305@126.com (J.L.); qyx20@mails.tsinghua.edu.cn (Y.Q.)
*Correspondence: hanwang@tsinghua.edu.cn.
[†]These authors contributed equally.

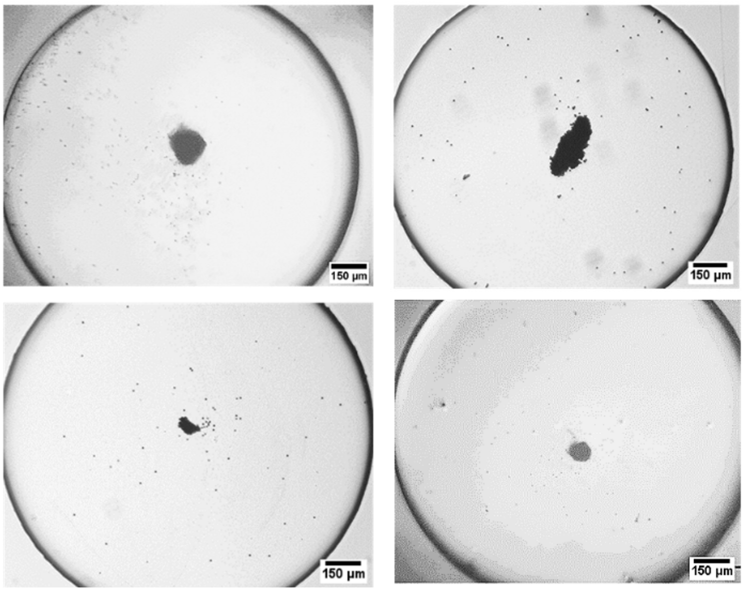

Figure S1. Microscopic images of the enriched microparticles in the microfluidic chambers.
